# Influence of Y-doped induced defects on the optical and magnetic properties of ZnO nanorod arrays prepared by low-temperature hydrothermal process

**DOI:** 10.1186/1556-276X-7-372

**Published:** 2012-07-07

**Authors:** Chung-Yuan Kung, San-Lin Young, Hone-Zern Chen, Ming-Cheng Kao, Lance Horng, Yu-Tai Shih, Chen-Cheng Lin, Teng-Tsai Lin, Chung-Jen Ou

**Affiliations:** 1 Department of Electrical Engineering, National Chung Hsing University, Taichung, 40227, Taiwan; 2 Department of Electronic Engineering, Hsiuping University of Science and Technology, Taichung, 41280, Taiwan; 3 Department of Physics, National Changhua University of Education, Changhua, 50007, Taiwan; 4 Department of Electrical Engineering, Hsiuping University of Science and Technology, Taichung, 41280, Taiwan

**Keywords:** Y-doped ZnO nanorods, Wurtzite, Saturation magnetization, Photoluminescence, Ferromagnetism

## Abstract

One-dimensional pure zinc oxide (ZnO) and Y-doped ZnO nanorod arrays have been successfully fabricated on the silicon substrate for comparison by a simple hydrothermal process at the low temperature of 90°C. The Y-doped nanorods exhibit the same *c*-axis-oriented wurtzite hexagonal structure as pure ZnO nanorods. Based on the results of photoluminescence, an enhancement of defect-induced green-yellow visible emission is observed for the Y-doped ZnO nanorods. The decrease of E_2_(H) mode intensity and increase of E_1_(LO) mode intensity examined by the Raman spectrum also indicate the increase of defects for the Y-doped ZnO nanorods. As compared to pure ZnO nanorods, Y-doped ZnO nanorods show a remarked increase of saturation magnetization. The combination of visible photoluminescence and ferromagnetism measurement results indicates the increase of oxygen defects due to the Y doping which plays a crucial role in the optical and magnetic performances of the ZnO nanorods.

## Background

The II-VI semiconductor zinc oxide (ZnO) with a direct wide bandgap (3.37 eV) and a large exciton binding energy (60 meV) has attracted substantial attention in the research community [1-3]. Although ZnO had been researched for the past decades, the renewed interests are focused on the low-dimensional nanostructures, such as nanoparticles [4], nanowires [5], nanarods [6], and nanotubes [7], due to brand new fundamental physical properties and applications of nanodevices. Progressive studies on the performance improvement of these one-dimensional nanostructured ZnOs for optoelectronic device applications have been performed by various growth methods, such as hydrothermal methods [8], vapor–liquid–solid [9], metal organic vapor-phase epitaxy [10], and pulsed laser deposition [11] and doped with impurities, such as Ag [12], Li [13], and P [14]. Besides, effective mass production process of ZnO nanowires by a modified carbothermal reduction method has been also reported [15]. Recent researches further denoted that nanostructured ZnO with large effective surface area is suitable for ultraviolet devices and photovoltaic applications, such as light-emitting diodes [16], nanolasers [17], photodetectors [18], field emitters [19], chemical sensors [20], and photo-electrodes in dye-sensitized solar cells [21].

Recently, the observation of ferromagnetism with high Curie temperature in III-V and II-VI semiconductors has also attracted a great deal of attentions [22-24]. Room temperature ferromagnetism of ZnO doped with transition metals has been also theoretically predicted and experimentally confirmed for spintronics applications [25,26]. In order to form diluted magnetic semiconductors, ZnO nanostructures have been doped with magnetic metal elements, such as Mn, Co, or Ni [27]. Recently, some researches declared that ferromagnetism had been obtained from undoped nanostructured ZnO and suggested to be induced by defects [28]. Non-magnetic elements, such Bi [29] or Li [30], have been doped into ZnO and room temperature ferromagnetism has been also observed. Therefore, ferromagnetism would not originate from the non-magnetic dopants since they do not contribute to ferromagnetism.

Based on the previous reports [1-30], one of the effective ways to approach the optical and magnetic properties of these nanostructured materials is the doping with selective elements. By choosing suitable rare-earth dopant, modification of the properties can be anticipated. In this present study, we will focus on the doping effect of larger non-magnetic element Y on the structural property of Y-doped ZnO (ZnO:Y) nanorods. In addition, the defect-related origin of optical and magnetic properties will be also discussed.

## Methods

The ZnO and ZnO:Y nanorod arrays were fabricated by hydrothermal method on the ZnO-seeded silicon substrate. Pure ZnO seed layers for both nanorod compositions were firstly deposited on silicon substrate by spin coating technique. Then, the source solutions for ZnO and ZnO:Y nanorods growth were prepared using the precursors, zinc acetate dihydrate Zn(C_2_H_3_O_2_)_2_2H_2_O and yttrium acetate hydrate Y(C_2_H_3_O_2_)_3_4H_2_O, in stoichiometric proportions within a blending solvent of de-ionized water and HMTA ((CH_2_)_6_ N_4_). Then, the seeded substrate was placed upside down into the solution contained in a closed vial at 90°C for 3 h to grow the nanorods. Finally, the samples were rinsed with de-ionized water and dried in air for characterization.

The crystal structure was determined by X-ray diffraction (XRD) spectrum using a Rigaku D/max 2200 X-ray diffractometer (Rigaku Corporation, Tokyo, Japan) with Cu-Kα radiation. Morphological characterization was observed using a field emission scanning electron microscope (FE-SEM, JEOL JSM-6700 F, JEOL Ltd., Akishima, Tokyo, Japan) at 3.0 kV. Secondary ion mass spectrometry (SIMS) was utilized to identify the elemental distribution. Room temperature photoluminescence (RTPL) spectroscope was used to measure optical emissions from 350 to 645 nm using the He-Cd laser with wavelength of 325 nm. Raman spectra of the samples were measured with an excitation wavelength of 532 nm from argon laser. Finally, the magnetization measurements were performed using a MicroMag™ 2900 alternative gradient magnetometer (AGM) (Princeton Measurements Corp., Princeton, NJ, USA) at room temperature to investigate the magnetic properties of ZnO and ZnO:Y nanorods.

## Results and discussion

**Figure 1:**
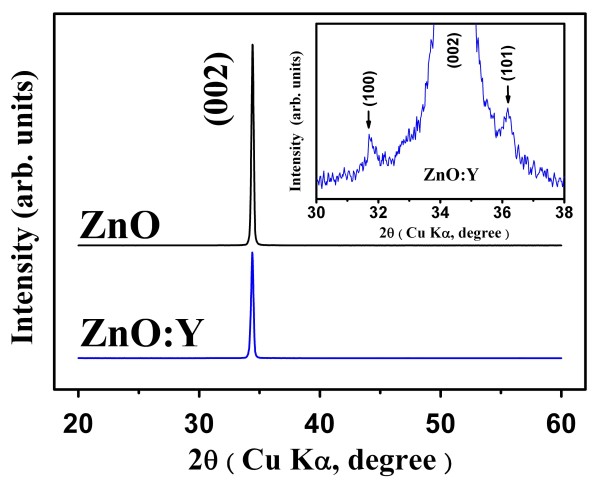
**X-ray diffraction patterns for ZnO and ZnO:Y nanorods.** The inset enlarges the patterns in 2*θ* region of 30 to 38.

Figure 1 illustrates the XRD spectra of ZnO and ZnO:Y nanorods. It is noted that ZnO:Y nanorods retain perfect (002) orientation and the same wurtzite hexagonal structure as the pure ZnO nanrods. The reactions of the ZnO nanorod formation synthesized by acetates and HMTA can be summarized as the following chemical formulas [31]:

(1) $${\left({\text{CH}}_{2}\right)}_{6}{\text{N}}_{4}+{6\text{H}}_{2}\text{O}\to 6\text{HCHO}+{4\text{NH}}_{3}$$

(2) $${\text{NH}}_{3}+{\text{H}}_{2}\text{O}\to {\text{NH}}_{4}{}^{+}+{\text{OH}}^{-}$$

(3) $${\text{Zn}}^{2+}+{2\text{OH}}^{-}\to \text{Zn}{\left(\text{OH}\right)}_{2}\to \text{ZnO}+{\text{H}}_{2}\text{O}$$

**Figure 2:**
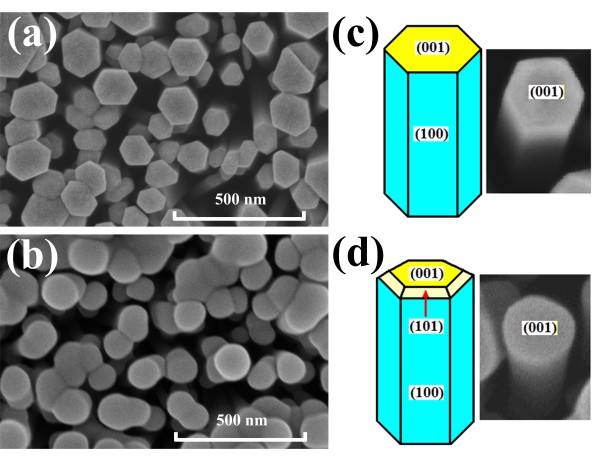
**SEM top-view and enlarged images.** SEM top-view image of (**a**) ZnO and (**b**) ZnO:Y nanorods and enlarged images of (**c**) ZnO and (**d**) ZnO:Y nanorods.

Under the hydrothermal conditions, HMTA will hydrolyze and release NH_3_ to provide (OH)^−^. Finally, the reaction of Zn^2+^ and (OH)^−^ brings the products, ZnO and H_2_O. It is obvious that HMTA plays a key role to form Zn-O bonds. The (002) plane of wurtzite-structured ZnO is terminated with Zn^2+^, resulting in polar top surfaces with positive charge. In the chemical solution, non-polar HMTA will precedently chelate the non-polar facets except the polar (002) plane for epitaxial method. Therefore, a preferential growth along (002) is reasonably observed. Meanwhile, substitution of Y for Zn during the growth of nanorods can be obtained, which is similar to the synthesized process of Ce-doped ZnO nanorods [32]. Compared with ZnO nanorods, the XRD spectrum of ZnO:Y nanorods exhibits obvious single diffraction peak of (002) and two slight peaks of (101) and (100) as shown in the inset of Figure 1. The obvious decrease of (002) diffraction peak intensity for ZnO:Y nanorods shows the restrain of the crystallization compared with ZnO samples. The result also indicates the suppression of growth rate along (002) crystal plane and slight enhancement of growth rate along (101) and (100) crystal planes. Besides, the decrease of *a*-axle lattice constants from 3.2526 to 3.2576 Å and *c*-axle lattice constant from 5.1849 to 5.1904 Å is obtained from the 2*θ* angles of diffraction peaks measured from ZnO and ZnO:Y nanorods. The reason is that the radius of Y^3+^ ion (0.92 Å) is larger than that of Zn^2+^ ion (0.74 Å), and the doping of Y into ZnO nanorods should lead the increase of *a*- and *c*-axis lattice constants and, correspondingly, the shift of all diffraction peaks towards lower 2*θ* angle.

(Figure 2a,b) shows the FE-SEM surface morphology images of the ZnO and ZnO:Y nanorods, respectively. The radius of ZnO:Y nanorods is slightly larger than that of ZnO which implied that the radial growth rate along <100 > directions is enhanced by the doping of Y. The enlarged single nanorod images of both compositions are shown in (Figure 2c,d) for comparison. Obviously, the blurred hexagon is observed for Y-doped ZnO due to a slight enhancement of growth rate along <101 > directions as indicated in (Figure 2c,d). The results coincide with the appearance of (100) and (101) peaks as shown in Figure 1.

**Figure 3:**
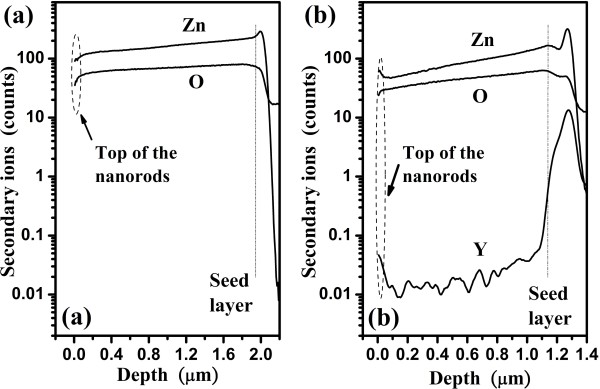
SIMS spectrum of the (a) pure ZnO and (b) ZnO:Y nanorods.

It has been reported that pure ZnO materials exhibit n-type semiconductor characteristics due to the natural existence of oxygen vacancies [33]. From the SIMS spectrum as shown in (Figure 3a,b), we obtain the O/Zn ratio decreased from 47% (ZnO) to 41% (ZnO:Y) which are recorded to examine the presence of oxygen vacancies between pure ZnO and ZnO:Y nanorods. The lesser counts of oxygen and zinc of ZnO:Y nanorods reveal the increase of defects with the introduction of Y in the nanorods. Hence, further analysis of RTPL and Raman shift spectra needs to be performed to figure out the Y doping effect on the origin of defects in the nanorods.

**Figure 4:**
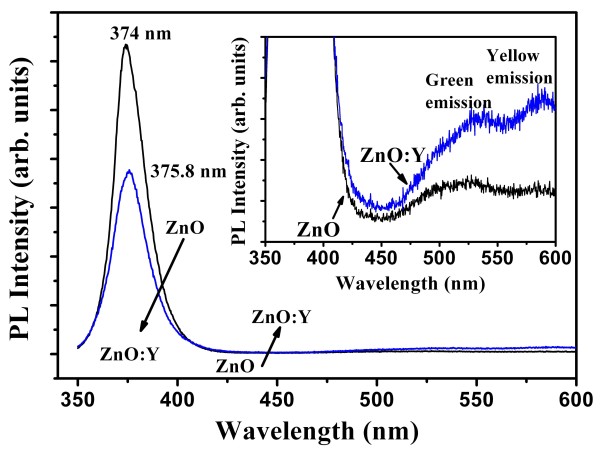
**PL spectra of the ZnO and ZnO:Y nanorods.** At room temperature with an excitation wavelength at 325 nm.

The RTPL spectra of the pure ZnO and ZnO:Y nanorods recorded with an excitation source from a 325-nm He-Cd laser are shown in Figure 4. In accord with that of the typical emission spectra of ZnO bulks and films, the RTPL spectra of both undoped and ZnO:Y nanorods are similar and are mainly composed of a strong ultraviolet emission and a broad green-yellow visible emission. The UV emission peak is originated from free excitonic recombination corresponding to the near-band-edge exciton emission of the wide bandgap ZnO through an exciton-excitation collision process [34]. The green-yellow visible emission, a deep-level emission, is attributed to the recombination of a photo-generated hole with an electron that belongs to an ionized intrinsic defects, depending on the fabrication process [35]. The green emission is generally attributed to the recombination of electrons trapped in single ionized oxygen vacancies (*V*_O_^+^) [36]. Besides, the yellow emission is related to double ionized oxygen vacancies (*V*_O_^++^) [37] or oxygen interstitials (O_*i*_) [38]. As shown in Figure 4, two luminescence emissions, a strong ultraviolet emission at 375.8 nm and a broad visible emission (including a splitting green emission at 532 nm and a splitting yellow emission at 584 nm) for both kinds of nanorods, were observed. As previously mentioned, the intensity of the ultraviolet emission is strongly dependent on the density of free excitons which is closely connected with the crystalline quality of ZnO and examined by previous reports [39,40]. Thus, for ZnO:Y nanorods, the lower crystalline quality results in the lower free exciton density and, consequently, the lower ultraviolet emission intensity [41]. The ratio of the emission intensities of visible to UV (*I*_Vis_/*I*_UV_) shows an increase from 0.0097 to 0.03078. The higher *I*_Vis_/*I*_UV_ ratio of ZnO:Y nanorods relative to ZnO nanorods indicates an increase of amorphous content and a restrain of crystallization for the ZnO:Y nanorods. Meanwhile, the enhancement of green and yellow emissions for ZnO:Y nanorods is also observed, which indicates the increase of intrinsic defects, *V*_O_^+^*V*_O_^++^, and O_*i*_. During the process of hydrothermal reaction, defects can be easily produced and, further, be increased by the doping of Y in the ZnO nanorods. It is worth noting that ZnO has a hexagonal close-packed lattice with a relatively open structure in which Zn atoms occupy half of the tetrahedral sites, and all the octahedral sites are empty. In general, oxygen vacancies (*V*_O_) have lower formation energy than the zinc interstitials (Zn_*i*_) and, therefore, should be more abundant in Zn-rich compositions for the real wurtzite ZnO structure [1]. As expected, intrinsic defects and extrinsic dopants can be easily introduced during fabrication process [42]. Therefore, by the doping of Y and corresponding increase of lattice constant, further open structure with more defects is a reasonable result.

**Figure 5:**
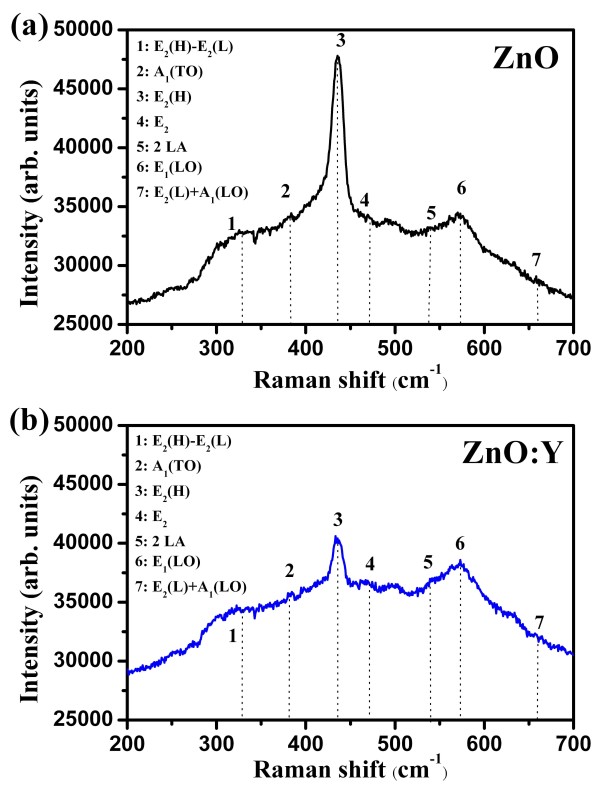
Raman spectra of the (a) ZnO and (b) ZnO:Y nanorods.

Raman scattering is another effective method to evaluate the crystallization and defects of nanostructure materials. In order to further investigate the influence of Y-doped induced defects on the optical and magnetic properties of ZnO nanorods, the room-temperature Raman spectra of ZnO and ZnO:Y nanorods were recorded from 200 to 700 cm^−1^ as shown in Figure 5. The ZnO nanorod exhibits hexagonal wurtzite structure with space group *P*6_3_mc, where all atoms occupy C_3*v*_ sites. Group theory predicts the Raman active zone centers of the optical phonons at the *Γ* point of the Brillouin zone to be A_1_(*z*) + E_1_(*x,y*) + 2E_2_[43]. The A_1_ and E_1_ are polar and infrared active modes and, hence, split into the transverse-optical (TO) and longitudinal-optical (LO) modes with different frequencies. The nonpolar E_2_ modes are Raman active only and have two frequencies, E_2_ high mode (denoted as E_2_(H)) and E_2_ low mode (denoted as E_2_(L)). For wurtzite ZnO structure, the typical peaks at 331, 384, 437, 467, 538, 573, and 660 cm^−1^ correspond to E_2_(H) − E_2_(L), A_1_(TO), E_2_(H), E_2_, A_1_(LO), E_1_(LO), and A_1_ modes, respectively [44,45]. As we can see in the Raman shift spectra as shown in Figure 5, a dominant E_2_(H) mode located at 437 cm^−1^ is observed for both compositions and shows good crystallization quality of wurtzite structure [46]. The band at 573 cm^−1^ is corresponding to E_1_(LO) mode. It is generally accepted that the E_1_(LO) is related to the formation of defects in the nanorods [47-49]. Therefore, the increase of E_1_(LO) intensity indicates the increase of defects including *V*_O_^+^*V*_O_^++^ and O_*i*_ in the ZnO:Y nanorods which is consistent with the result of PL spectra. Furthermore, the intensity ratio of the E_2_(H) peak to E_1_(LO) peak shows a decrease from 1.39 to 1.06. The decrease of the ratio reveals the restraint of crystallization, which coincide with the lower diffraction peak intensity in XRD pattern and *I*_Vis_/*I*_UV_ ratio in RTPL spectrum of ZnO:Y nanorods.

**Figure 6:**
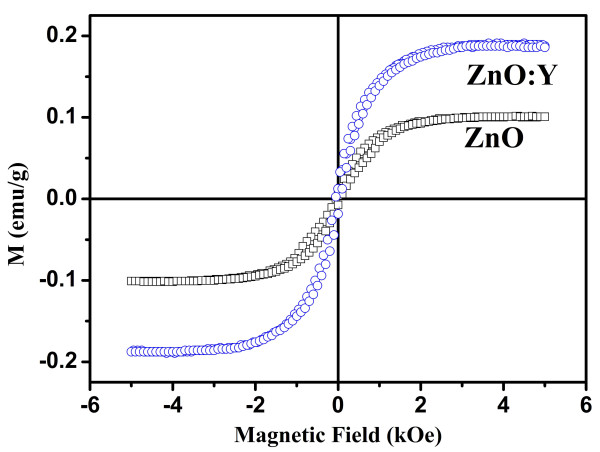
Magnetization curve measured at room temperature of the ZnO and ZnO:Y nanorods.

The magnetization curve measurements were performed using an AGM with an external magnetic field between −5 and 5 kOe at room temperature. As shown in Figure 6, results of magnetic characterization show obvious ferromagnetic behavior for both compositions. Besides, the increase of saturation magnetization is observed from 0.101 emu/g for ZnO nanorods to 0.189 emu/g for ZnO:Y nanorods. The enhancement of ferromagnetism for the nanorods originates from the increase of defects including oxygen vacancies and interstitials, which can be clarified with the bound magnetic polaron model proposed by Coey et al. [50]. The exchange interactions that couple the individual moments can be explained by electrons locally trapped by defects magnetized by external magnetic field which is the origin of ferromagnetism. Liu et al. [37] studied the magnetic defects by first-principle calculations and showed that the local magnetic moments for the *V*_O_^+^ and O_*i*_ are 0.98 and 2.00 μ_B_, whereas *V*_O_^++^ is 0 μ_B_. As the results from RTPL and Raman spectra, oxygen vacancies and interstitial are increased by Y doping. Therefore, the saturation magnetization increases with the doping of Y into the ZnO nanorods. Therefore, combined with the increase of visible emission intensity in RTPL spectra, E_1_(LO) peak intensity in Raman shift spectra, and saturation magnetization in magnetization curves, the increased defects are responsible for the origin of the enhanced RT ferromagnetism by the introduction of Y in the ZnO nanorods.

## Conclusions

ZnO and ZnO:Y nanorods have been successfully synthesized at a low temperature by hydrothermal method. The correlation between the Y-doped induced defects in the ZnO:Y nanorods and their structural, optical, and magnetic properties was studied in details. It was found that the doping of Y results in the increase of defects which also affects the corresponding structural, optical, and magnetic properties of the nanorods. The XRD spectra show that crystallization is suppressed by the doping of Y in the nanorods. The increase of visible emission in RTPL spectra and E_1_(LO) intensity in Raman spectra demonstrates that the doping of Y will increase the doping-induced defects in the nanorods. Magnetization curve measurements show the room temperature ferromagnetism of both nanorods. Finally, the combination of the optical and magnetic measurement results reveals that the oxygen defects play a crucial role in introducing ferromagnetism which can be enhanced by the doping of Y in the ZnO nanorods.

## Competing interests

The authors declare that they have no competing interests.

## Authors’ contributions

S-LY and C-YK conceived of the study, participated in its design and coordination, as well as in the discussions and data analysis, and wrote the manuscript. C-CL and T-TL carried out the nanorod formulation experiments, contributed to data interpretation, and helped in writing the manuscript. LH and Y-TS participated in the design of the study, carried out the PL, Raman spectra, and AGM experiments. H-ZC and M-CK analyzed and organized the results. C-JO has been involved in revising the manuscript critically. All authors read and approved the final manuscript.

## Authors’ information

C-YK is a professor of the Department of Electrical Engineering, National Chung Hsing University. S-LY, H-ZC, and M-CK are professors of the Department of Electronic Engineering, Hsiuping University of Science and Technology. LH and Y-TS are professors of the Department of Physics, National Changhua University of Education. C-CL and T-TL are Ph.D. students. C-JO is a professor of the Department of Electrical Engineering, Hsiuping University of Science and Technology.
